# The clinical and genetic features of patients with hyper-immunoglobulin D syndrome (HIDS)

**DOI:** 10.1186/1546-0096-12-S1-P257

**Published:** 2014-09-17

**Authors:** Betul Sozeri, Ozlem Cam, Metin Delebe, Sevgi Mir, Afig Berdeli

**Affiliations:** 1 Pediatric Rheumatology, Ege University Faculty of Medicine, Izmir, Turkey; 2 Pediatrics, Ege University Faculty of Medicine, Izmir, Turkey; 3 Moleculer Genetic, Ege University Faculty of Medicine, Izmir, Turkey

## Introduction

Mevalonate kinase deficiency (MKD) is a rare autosomal recessive disorder causing 1 of 2 phenotypes, hyper-immunoglobulin D syndrome and mevalonic aciduria, presenting with recurrent fever episodes, often starting in infancy, and sometimes evoked by stress or vaccinations. This autoinflammatory disease is caused by mutations encoding the mevalonate kinase (MVK) gene and is classified in the group of periodic fever syndromes. HIDS is characterized by recurrent fever attacks of 3-7 days that begin in infancy and recur every 4-6 weeks. The febrile period is accompanied by lymphadenopathy, arthralgia, abdominal pain, diarrhea, aphthous ulcers, and varying degree of skin involvement. The course and severity of the disease may be quite different. There is no effective or proven therapy for HIDS.

## Objectives

We aim to determine the clinical and genetic characteristics together with the underlying MVK genotypes in single center during a period of 2 years.

## Methods

A retrospective review of medical records for patients referred for HIDS over 2 years. We obtain 40 patients (22 female, 18 male) and 25 healthy controls.

## Results

The median age of first attack was 33 months (range 2-62 months). The median age of diagnosis was 60 months. The most common symptoms of high fever accompanied respectively: lymphadenopathy (n=27), abdominal pain (n=26), arthralgia (n=20), diarrhea (n=15), aphthous stomatitis (n=13), vomiting (n=12) and maculopapuler rash (n=6) were determined. Amyloidosis was found in a patient (0.3%). The mean serum IgD level was 129±92 mg/dl, it was be normal in 43% of patients.

We found mutations in 75% of patients (n=30) in exon 3 and 11 (c155G>A; c1129G>A) in MVK gene. We applied colchicine therapy in 40% of patients while intermittent steroid therapy 20% of patients. Empiric colchicine (40%) and glucocorticosteroids (20%) controlled flares in majority of patients with HIDS. Three patients had also tonsillectomy. We used biologic therapy in 3 patients ( canacinumab (n=2), anakinra (n=1)).

## Conclusion

In conclusion, HIDS is characterized with early onset an autoinflammatory disease and may to result frequent and uncontrolled attacks.

## Disclosure of interest

None declared.

